# Control of *Urochloa decumbens* Using Glyphosate Applied by Remotely Piloted Aircraft and Ground Sprayer with Different Spray Nozzles

**DOI:** 10.3390/plants13060757

**Published:** 2024-03-07

**Authors:** Luana de Lima Lopes, João Paulo Arantes Rodrigues da Cunha, Quintiliano Siqueira Schroden Nomelini, Cleyton Batista de Alvarenga

**Affiliations:** 1 Institute of Agrarian Sciences, Federal University of Uberlândia, Uberlândia 38408-100, Brazil; luana.lopes@ufu.br (L.d.L.L.); cleytonalvarenga@ufu.br (C.B.d.A.); 2 Math College, Federal University of Uberlândia, Uberlândia 38408-100, Brazil; quintiliano.nomelini@ufu.br

**Keywords:** *Brachiaria*, control effectiveness, weeds, crop protection, application quality

## Abstract

The use of remotely piloted aircraft (RPA) to spray pesticides currently occurs, but knowledge about this technology is lacking due to the different locations, targets, and products applied. The objective of this study was to evaluate the control of *Urochloa decumbens* with glyphosate applied using an RPA (10 L ha^−1^) equipped with different spray nozzles (XR 11001 and AirMix 11001). For the purpose of comparison, ground application was also performed (100 L ha^−1^). The deposition was evaluated by means of the quantification of a tracer by spectrophotometry, the droplet spectrum was evaluated with water-sensitive paper, and the control efficiency was evaluated based on visual measurements with percentage scores. Statistical process control was used to analyse the quality of the deposition in the area. The results showed that the application via RPA presented a greater amount of tracer on the leaves than the ground application, suggesting that the former is a good option for application, even providing a lower coverage and number of droplets per area. Both application methods were effective at controlling *Urochloa decumbens*. The nozzles showed potential for use in applications, with control efficiency higher than 84% from 21 days after application. The percentage of droplets smaller than 100 μm in the applications was less than 5%. No nonrandom behaviour was observed during deposition, indicating a high-quality process.

## 1. Introduction

Signal grass [*Urochloa decumbens* (Syn. *Brachiaria decumbens*)] is a perennial grass native to tropical Africa [1]. It is considered a highly relevant weed species in agricultural crops due to its high adaptability and management challenges. In agricultural cultivation areas, these plants can cause significant problems because they compete for water, light, space, and nutrients, in addition to acting as hosts for pests and pathogens common to crops and intervening in the harvest [2,3].

In order to control this pest, the application of herbicides is predominantly used [4] based on the control cost and efficiency relationship [5]. Glyphosate [N-(phosphonomethyl)glycine] is one of the most widely used herbicides, with broad-spectrum systemic action [6,7]. It is composed of phosphonate and inhibits the shikimate pathway by means of the enzyme 5-enolpyruvylshikimate-3-phosphate synthase (EPSPs) [8,9], interfering with the biosynthesis of aromatic amino acids [10]. It is a nonselective herbicide for conventional crops that have not been genetically modified to be tolerant [11], with postemergence application of the target plant.

The effectiveness of pesticides for controlling pests strongly depends on whether the desired amount of product reaches the target plants. Thus, the spraying system has a great impact on the efficiency and effectiveness of the application [12]. One new strategy is the use of remotely piloted aircraft (RPA) for the application of pesticides. An RPA is an unmanned aircraft consisting of a flight deck, navigational flight control, and a spraying mechanism. These devices can be operated manually by remote control or automatically by a global positioning system (GPS) with preplanned flights [13].

RPA have great potential for covering areas that are difficult to access with ground spray machines and manned aircraft [14]. The use of RPA in pesticide applications has achieved good results in terms of pest control, spraying accuracy, distribution uniformity, and ease of operation [15]. Furthermore, as the position of the RPA exceeds the maximum height of the crop, mechanical damage to the crop should be minimal [16].

Rotary-wing RPA have good manoeuvrability, vertical take-off and landing capacity, and as they are remotely controlled, they eliminate problems related to operator exposure to products [17]. During application, the droplets are deposited below the aircraft under the influence of the airflow descending from the rotor [18]. This airflow (downwash) is defined as the deflection of the air stream as a function of the aerodynamic action of the propeller blades. It is part of the process of maintaining the altitude of the RPA, and it is dependent on RPA characteristics [19]. Cavalaris, Karamoutis, and Markinos [20] asserted that the number of rotors, motor power, rotor design, and position and type of spray nozzles used can affect the effective range of application and droplet deposition on the target. The effective range refers to the width of the range where there is uniform distribution of the application considering the overlaps of other passes.

The correct deposition of the spray solution on the target depends on the droplet size distribution provided by the spray nozzles [21] and wind speed and direction. Spray nozzles are designed to control the characteristics and direction of the solution spray [22]. Flat fan nozzles are commonly used in many applications and may feature an extended range, air induction, and pre-orifice. Extended range nozzles are frequently used because these nozzles can work over a wide pressure range and have finer droplet sizes, which correlates with better droplet retention by the target [23] because finer droplets may provide better target coverage [24].

The droplet size affects the quality of the spray solution, in addition to the loss due to drift. A reduction in the number of fine droplets may decrease the potential for exodrift [25]. In this sense, air induction nozzles can be used. These nozzles can produce larger droplets than others without air induction at the same flow rate due to the presence of microbubbles, resulting in reduced drift [26]. However, there is a lack of research on control effectiveness promoted by applications with RPA using air induction nozzles. Therefore, studies are necessary to verify the performance of this application and to compare it with that of conventional nozzles [27].

Thus, due to the need to better understand this new technology, the present study aimed to evaluate the control of *Urochloa decumbens* with glyphosate herbicide applied using an RPA (10 L ha^−1^) equipped with different spray nozzles (XR 11001 and AirMix 11001). For the purpose of comparison of the performance of the technique, ground application equipment was also used (100 L ha^−1^).

## 2. Results and Discussion

### 2.1. Deposition

Table 1 shows the median deposition of the sprayed herbicide solution. Greater deposition was achieved with remotely piloted aircraft (RPA) (3.466 μg cm^−2^) than with ground application via a backpack sprayer pressurized with CO_2_ (2.242 μg cm^−2^). There were no significant differences for the different nozzles used in the application. The XR 11001 yielded a median deposition of 2.919 μg cm^−2^ and the AirMix 11001 yielded 2.372 μg cm^−2^. Jeevan et al. [28] observed similar behaviour. The authors explain that applications via RPA showed higher deposition due to the reduced application rate, with a higher concentration of the active ingredient in the droplets and lower runoff to the soil surface. Other authors observed that the downwash effect had a positive influence on deposition, in which downward pressure on the droplets favoured deposit and decreased drift [29,30]; these observations may explain what occurred in the current study.

It is important to remember some points. Low-volume applications require a more efficient deposition of droplets on the target surface [31] due to the use of more concentrated solution and the volume distributed over the area, so that there is no loss of product, reduced effectiveness, or increased environmental risks. The deposition characteristics may be different for different RPA models. Sinha et al. [32] found that although nozzles located under each rotor are quite common in commercial RPA, nozzles arranged in a boom yielded a lower drift potential. The authors attribute this performance to the aerodynamic differences for the nozzle arrangements in relation to the rotors of the RPA.

**Figure 1:**
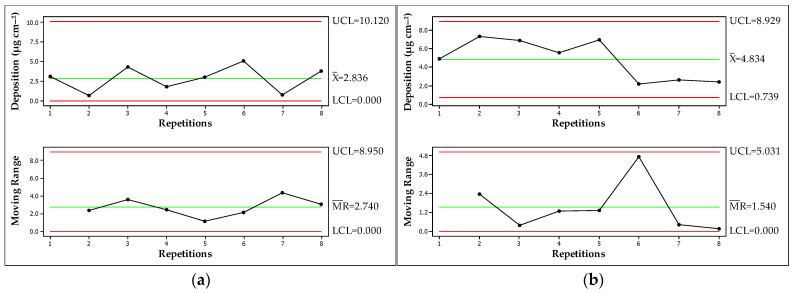

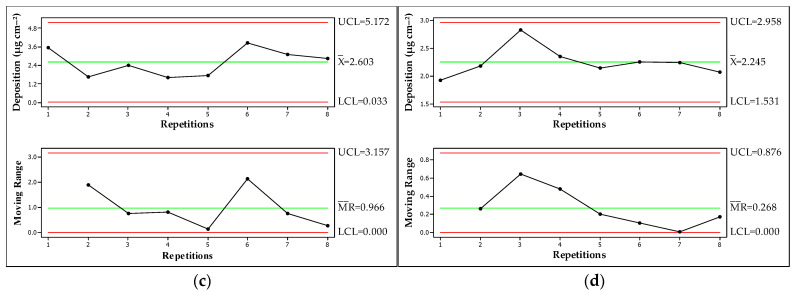
Control charts for tracer deposition (μg cm^−2^) during the application of herbicide (glyphosate) for the control of *Urochloa decumbens* using an RPA and a backpack sprayer with two different spray nozzles. (**a**) RPA—XR 11001; (**b**) RPA—AirMix 11001; (**c**) GROUND—XR 11001; (**d**) GROUND—AirMix 11001. UCL: upper limit, $\bar{X}$: treatment mean, LCL: lower limit, $\bar{MR}$: moving averages; RPA: remotely piloted aircraft.

Control charts are tools employed to analyse whether or not a process is under statistical process control and they are commonly used to monitor undesirable changes [33]. Despite the technological advances in application technology, the spraying process may exhibit deficiencies, so it can be beneficial to obtain application data in the area. Deposition analysis through control graphs can show how well the deposition is controlled, as shown in Figure 1.

The graphs show that applications via RPA (Figure 1a,b) showed greater variation in relation to the average value. Application via RPA with the XR 11001 nozzle (Figure 1a) presented, in replicate six, a deposition of 5.128 μg cm^−2^, while the overall mean value of the treatment was 2.836 μg cm^−2^, constituting a variation of 2.292 μg cm^−2^. When the application was performed with the AirMix 11001 nozzle (Figure 1b), for the same sprayer, the greatest variation was 2.659 μg cm^−2^; in this treatment, the mean was 4.834 μg cm^−2^ and in replicate six, the deposition was 2.175 μg cm^−2^.

The ground application resulted in less deposition variation in relation to the mean value; that is, the data for this application method tended to be closer to the mean value of the treatment. When the application was performed with the XR 11001 nozzle (Figure 1c), the largest difference (1.273 μg cm^−2^) occurred between the sample (replicate six, 3.876 μg cm^−2^) and the mean (2.603 μg cm^−2^). For application with the AirMix 11001 nozzle (Figure 1d), replicate three presented a deposition of 2.825 μg cm^−2^, and the treatment mean was 2.245 μg cm^−2^, with a variation of 0.580 μg cm^−2^. The variation between repetition six (2.245 μg cm^−2^) and the mean (2.245 μg cm^−2^) was equal to zero, with no variation between the evaluated point and the treatment mean.

Although the results showed variations in application, the process was well controlled for all treatments according to the statistics obtained. As seen in the graphs; the data are all positioned between the upper (UCL) and lower (LCL) limits, indicating a quality application from the point of view of the distribution of the spray in the area. There were no patterns of nonrandomness. A process is out of control when points in the control chart are outside the upper or lower limits. Thus, the process changes from an average with projected variation over the sampling [34]. High variability in deposition may be responsible for excessive or insufficient product application within the applied area. Process analysis facilitates intervention and corresponding changes in process parameters for better performance [35].

Notably, although the results reveal that the process was under control, the values obtained are not necessarily the desired values. The control graphs show that the ground application yielded less variation but also lower mean values of deposition. Jorani et al. [36] state that the detection metrics of the X bar graph (the one used in this study) only take information about new data samples to the decision-making process and ignore other information, such as ideal deposition values.

### 2.2. Droplet Spectrum

Table 2 shows the average coverage (%), droplet density (droplets cm^−2^), and relative amplitude (RA) obtained by the application of the herbicide spray (glyphosate). The coverage, which represents the proportion of the area occupied on the surface of the target by the droplets, was greater for the ground application, at 18.1%. Application via RPA presented 5.1% coverage. There was no significant difference in coverage with the different nozzles. The XR 11001 standard flat fan showed 10.7% coverage, and the coverage of the AirMix 11001 air induction flat fan was 12.5%. As expected, an increase in the application rate provided an increase in coverage. It should be noted that higher application rates may pose the risk of runoff from the deposition area (grass), although it offers a greater probability of target coverage [37].

Subr, Al-Ahmadi, and Abbas [38] reported that a standard flat spray nozzle (12003 Agroplast ^®^, Sawin, Poland) produced greater coverage than an air induction nozzle (8MS 03C Agroplast ^®^, Sawin, Poland), with 11.5% and 5.9% coverage, respectively, for application to eggplant (*Solanum melongena* L.) via a pressurized backpack sprayer (284 L ha^−1^). Ya, Yu, Kang, and Lee [27] evaluated application via RPA for rice (*Oryza sativa* L.) and soybean (*Glycine max* (L.) Merrill) plants with air induction flat fan (AI) nozzles designed by the authors and standard flat fan spray nozzles (XR 110015VS). Regarding coverage, values of 5.1% and 3.4% for the AI nozzle and 2.1% and 1.4% for the XR 110015VS nozzle were obtained for rice and soybean, respectively. The larger droplet size produced by the AI apparently reduced the drift, increasing the number of droplets that reached the target, thus increasing the coverage. Abdelmotalib et al. [39] reported that when the droplets generated by air induction nozzles impact the target surface, they are fragmented into smaller droplets, which reduces the drift potential and allows for better controlled application, which may improve coverage.

The density, which represents the number of droplets in an area, was higher for the method with the higher rate (100 L ha^−1^), ground application via a backpack sprayer, with 239.9 droplets cm^−2^; application via RPA presented a density of 55.4 droplets cm^−2^. This effect has also been observed by different researchers. Chen et al. [40] performed applications of thidiazuron and ethephon solution for cotton (*Gossypium hirsutum* L.) defoliation via RPA at different application rates (15, 18, 22.5, 25.5, 29.1, and 30 L ha^−1^) and observed that, as the volume of spray solution increased, the average droplet density gradually increased, with a mean of approximately 17 droplets cm^−2^ at the lowest rate and 27 droplets cm^−2^ at the highest rate. Önler et al. [41] also observed that the density increased as the application rate increased. For the different nozzles used, application with the standard flat fan XR 11001 yielded the highest droplet density, with 213.9 droplets cm^−2^, which can be attributed to the smaller droplet size. The flat fan nozzle with air induction yielded 81.4 droplets cm^−2^. This can be explained by the findings of Mur et al. [42] that smaller droplets are more likely to be deposited on the target than droplets of the same volume distributed in larger droplets. Considering the indications of Syngenta Crop Protection AG (Basel, Switzerland), which recommends at least 30–40 droplets cm^−2^ for postemergence herbicide applications [43] and evaluating only the density found for the different nozzles in this study, both have the potential to facilitate efficiency in weed control. Urach Ferreira [44], evaluating applications with the herbicide pyroxasulfone, found that higher droplet densities did not result in better weed control efficacy.

Note that the values of coverage and droplet density should be viewed with caution because there are no standardized ranges of values that inform the ideal percentage of coverage nor the number of droplets cm^−2^ for each active ingredient correlated with biological efficacy. There are indications [37], as previously discussed, but efficacy studies are important to better understand the disease, pest, or weed control.

The RA indicates the homogeneity in the droplet size [45]. The lower the RA value is, the lower the amplitude between the values of the diameters of the droplets produced during spraying and, consequently, the better the quality of the application, as the process is more easily controlled. In this study, the RA was 1.0 for the application via RPA, denoting lower dispersion in the droplet spectrum, indicating that the uniformity of the droplet size distribution was greater with this application method. The ground application presented an RA of 1.3. For the different nozzles used in the application, XR 11001 and AirMix 11001, there was no significant difference in RA. Xue et al. [46] suggest that RA values lower than one (1) indicate good homogeneity in droplet size distribution, as was found for the application via RPA. A spray with a small RA is important to increase application efficiency, as there will be a greater number of droplets sized according to the target’s needs [47].

Researchers [48,49] have shown that the herbicide glyphosate added to water spray has the ability to reduce the surface tension of fluid, and Xue et al. [50] reported that decreasing the surface tension of the spray solution can inhibit its shape retention capacity and promote its rupture, modifying the droplet spectrum and leading to a more uniform size distribution. This may have occurred in the present study, where the application at a rate of 10 L ha^−1^ yielded a higher concentration of herbicide and greater homogeneity than the spray solution of 100 L ha^−1^. This higher concentration may have a greater effect on the physical characteristics of the spray solution, resulting in greater alteration in the droplet spectrum.

Table 3 shows the means of the volume median diameter (VMD) and the percentage of droplets smaller than 100 μm, where the interaction between the method and nozzle factors was significant for these variables. The AirMix 11001, which introduces air into the droplets, exhibited higher VMD values regardless of the application method, with average values of 393.4 and 436.0 μm for applications via RPA and the backpack sprayer, respectively, than the XR 11001. Furthermore, this same nozzle (AirMix 11001) had a lower VMD value for application via RPA (393.4 μm) than for ground application (436.0 μm), demonstrating that this type of nozzle may be more influenced by the different application methods.

Gibbs, Peters, and Heck [51] observed that, compared with ground sprays, applications via RPA produced smaller droplets. This may occur due to a secondary breakdown of the droplets promoted by the air flow from the blades. Furthermore, during the droplet formation process, when air is incorporated into the droplet, the bubbles inside the droplets form weaker points that facilitate disintegration [52,53]. This may explain what occurred in this study when the AirMix 11001 nozzle was used.

Note that in herbicide applications, especially by aerial methods, the droplet size should be chosen very carefully. There is a compromise between the greatest effectiveness, sometimes achieved as a function of better leaf coverage using smaller droplet sizes, and minimizing the risk of herbicide exodrift, which is achieved using larger droplet sizes [54]. However, if the droplet diameters are very large, the spray is not uniformly deposited on the target, which may favour runoff due to the action of gravity [55]. Butts et al. [56] showed that droplets sized 395 μm maximize weed mortality when a dicamba and glyphosate herbicide mixture is applied at 94 L ha^−1^ and that the droplet size can be increased to 620 μm while maintaining 90% weed control and mitigating the potential drift risk, suggesting the use of air induction nozzles.

The drift potential, expressed as the percentage of droplet volume smaller than 100 μm, was greater for applications with the standard XR 11001 flat fan nozzle (2.4% and 4.1%) than for those with the AirMix 11001 flat fan with air induction (0.8% and 0.5%) for the different application methods. This presents reductions of 66.8% and 86.8% for aerial and terrestrial applications, respectively, with an air induction nozzle, when compared to the application with the standard flat fan nozzle. Therefore, the air induction nozzle presented fewer smaller droplets, resulting in a lower exodrift potential; thus, these droplets were less likely to deviate from the target area or evaporate into the atmosphere. The application with the XR 11001 had a greater percentage of droplets smaller than 100 μm for application via a ground sprayer (4.1%) than for application via RPA (2.4%). This may have been due to the droplets being closer to the value of the VMDs found in the application via RPA, which were above 200 μm. Despite the difference between some treatments, notably, the percentage of droplets smaller than 100 μm did not reach 5%. When the AirMix 11001 was used, there was no significant difference in the volume of droplets smaller than 100 μm for the different application methods.

Feng et al. [57] observed that downwash may result in a more uniform and regular spray distribution. Notably, the droplets sprayed by a hydraulic nozzle, under the influence of downwards airflow, reach a certain speed that can result in the droplets reaching the water-sensitive paper, producing a trail that can increase the identified droplet size. Moreover, measuring droplet sizes less than 50 μm is not feasible for this methodology, so the identified droplet spectrum may be increased [58]. This may also have contributed to the lower percentage volume of droplets smaller than 100 μm in the application via RPA in this study.

### 2.3. Effectiveness in Controlling Urochloa Decumbens

Means and medians of the percentage of effectiveness in controlling *Urochloa decumbens* can be seen in Table 4. There was no difference in the control effectiveness between the application methods, RPA and ground. At 14 days after application (DAA), the control means were 77.2% and 75.5%, respectively; at 21 DAA, the control medians were 93.0% and 95.0%, respectively; and at 28 DAA, the control medians were 90.0% and 88.0% for application via RPA and ground, respectively. This finding is interesting because the use of a reduced spray volume, without loss of control effectiveness, may result in cost reduction [59]. Jeevan et al. [28] evaluated the effect of different herbicide application rates for weed control in transplanted rice. For applications via RPA, rates of 15, 20, and 25 L ha^−1^ were studied, and for ground application with a manual backpack sprayer, a rate of 500 L ha^−1^ was used. Based on the results, the authors concluded that the application of 25 L ha^−1^ via RPA provided better weed control efficiency and higher crop yield, possibly due to better deposition.

Compared with the AirMix, the XR11001 had greater weed control effects on all the days evaluated, with 78.1% weed control at 14 DAA, 95.0% weed control at 21 DAA, and 90.0% weed control at 28 DAA. The AirMix 11001 showed a weed control of 74.6% at 14 DAA, 90.0% at 21 DAA, and 85.0% at 28 DAA. Ferguson et al. [60] reported that some herbicides are less effective when sprays have larger droplets because these droplets may not be deposited on the target plant, especially for target plants with small and narrow leaves. Research related to the pesticide application technology suggests that droplets sized 100 to 300 μm are suitable for herbicide spraying [61], as found for the XR 11001 in the current study. However, notably, from 21 DAA, both nozzles showed satisfactory control, above 84%.

Wang et al. [62] observed that there was no significant difference for the biological efficacy in weed control in wheat crops for different standard flat spray nozzles and air-induced flat spray nozzles. In all cases, the efficacy values were greater than 80%. The authors explained that although fine droplets tend to improve the adhesion and density of droplets on targets, they also have greater potential for drift, which may reduce deposition on targets.

**Figure 2:**
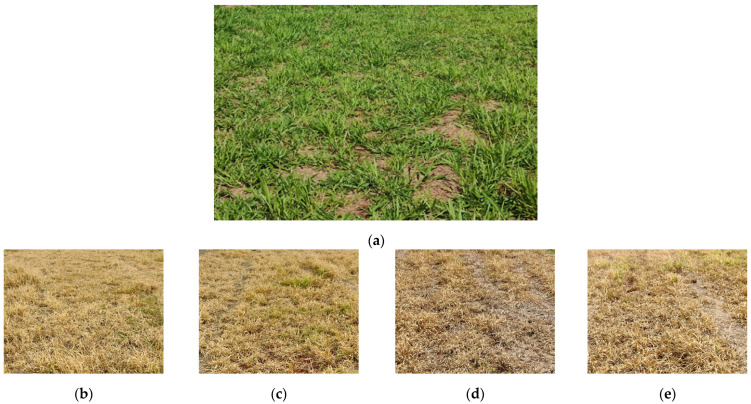
Control efficacy of the herbicide mixture (glyphosate) on *Urochloa decumbens* (%) at 21 DAA as a function of different application methods and with the use of different nozzles. (**a**) Area without herbicide spray application; (**b**) RPA—XR 11001; (**c**) RPA—AirMix 11001; (**d**) GROUND—XR 11001; (**e**) GROUND—AirMix 11001. DAA: days after application; RPA: remotely piloted aircraft.

In this study, after 21 DAA, the weed control was acceptable or very good, with values equal to or greater than 85% (Table 4). The image of the control efficiency of *Urochloa decumbens* (%) at 21 DAA of the herbicide mixture (glyphosate) can be seen in Figure 2 for the different treatments studied. At 14 DAA, there was possibly not enough time for the activity of the herbicide molecule to be complete based on plant metabolism. Brightenti et al. [63] observed that glyphosate application had a control efficiency of 85% at 25 DAA for *Urochloa decumbens*. Silveira et al. [64] found 100% control of *Urochloa decumbens* at 15 DAA, and the authors explained that in the early stages of development of these grasses, control is facilitated because the plant tissues have a greater capacity to transport herbicide molecules and have a lower capacity to recover from the injuries caused. In the aforementioned study, pesticide application was carried out when the plants had three to four formed tillers (new grass shoots) and an average height of 0.30 to 0.35 m.

## 3. Materials and Methods

### 3.1. Experiment Location

The field experiment was conducted during the summer season, and the applications were performed at the Experimental Farm of the Glória Campus of the Federal University of Uberlândia (UFU), with the latitude 18°56′51″ S and longitude 48°12′02″ W, in the city of Uberlândia, Minas Gerais, Brazil, during the month of March 2023 (all the applications were conducted on 8 March 2023). The area has an average altitude of 935 m, and the characteristic climate of the region is megathermic humid tropical Aw according to the Köppen classification, characterized by a hot and humid summer season and a cold and dry winter season. The average monthly air temperature ranges from 20.9 to 23.1 °C. The average annual rainfall is 1500–1600 mm [65].

The field where the experiment was conducted was an area infested with *Urochloa decumbens.* The plants exhibited a homogeneous phenological stage and had an average height of 0.25 m on the day of application.

### 3.2. Experimental Unit, Equipment, and Treatments

**Figure 3:**
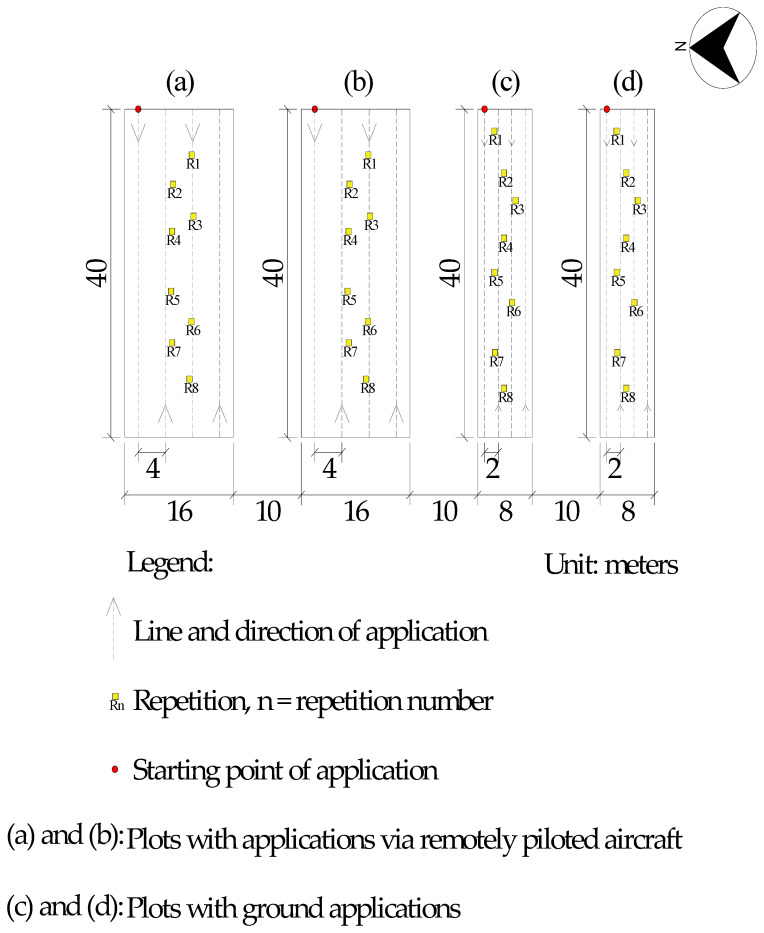
Experimental arrangement.

The area in which the applications were conducted (Figure 3) consisted of four plots. Where herbicide spray applications were conducted via a remotely piloted aircraft (RPA), the plots were 40 m long and 16 m wide, totalling an area of 640 m^2^. For sampling, 5.0 m strips were disregarded on each of the lateral edges and ends. The working width for aerial application was 4 m, based on the study conducted by Cunha e Silva [66]. The plots that received applications with the backpack sprayer pressurized with CO_2_ were 40 m long and 8 m wide, totalling 320 m^2^. In the plots, 1.0 m strips at each edge were disregarded to eliminate border effects. The working width was 2 m (boom width). The borders ensured that the speed of the sprayers was constant in the area analysed. The distance between the plots was 10 m (buffer zone).

The distribution of repetitions can be seen in Figure 3. When conducting an experiment with RPA (where the plots are usually larger in area), it is not possible to perform the repetitions in blocks. So, each experimental plot had a treatment with an application method and a type of nozzle. Biglia et al. [67] stated that, in this way, it is possible to minimize the variation in environmental and local conditions between repetitions, making the data obtained comparable. It also allows for the application of statistical process control, as described later.

Agricultural sprayers of the remotely piloted aircraft (RPA) type AGRAS MG-1 (DJI, Shenzhen, China) were used for the application, with a 10 L sprayer tank, 4 spray nozzles, and 8 motors (130 rpm/volts), as found in Table 5. The backpack sprayer was pressurized with CO_2_, equipped with a boom with four nozzles spaced 0.5 m apart.

The nozzles used in the application were an XR 11001 standard flat fan from Teejet^®^ Technologies (Glendale Heights, IL, USA) and an AirMix 11001 air induction flat fan from Agrotop^®^ Spray Technology (Obertraubling, Germany).

For application via RPA, the flight speed was 3.28 m s^−1^, the application rate was 10 L ha^−1^, and the altitude was 2.0 m. In ground spraying, for both nozzles, the working pressure was 200 kPa, and the flow rate of each nozzle was 0.33 L min^−1^. The displacement speed was 1.11 m s^−1^, the application rate was 100 L ha^−1,^ and the working height, relative to the crop, was 0.5 m.

During the experiment, the weather conditions in the area presented relative humidities ranging from 47.0% to 76.0%, with a mean of 66.3%, and air temperature ranging from 28.0 to 31.0 °C, with an average of 29.6 °C. Additionally, the wind speed ranged between 0.39 and 1.72 m s^−1^, with an average of 1.05 m s^−1^, as measured by a digital Thermohygro-anemometer portable model KR825, from AKROM^®^ (São Leopoldo, Brazil).

The experiment used a completely randomized design (CRD) with two factors (nozzle and application method), for a total of 4 treatments and eight replicates. 

### 3.3. Evaluations

#### 3.3.1. Deposition

The deposition on *Urochloa decumbens* was quantified by adding a tracer consisting of food colouring Blue Brilliant (FD&C Blue n. 1) internationally catalogued by Food, Drug & Cosmetic to the mixture at a dose of 0.5 kg ha^−1^. The herbicide added to the spray solution was Roundup Transorb^®^ R glyphosate from Bayer Crop Science (Leverkusen, Germany) based on the potassium salt of N-(phosphonomethyl) glycine (588 g L^−1^) and acid equivalent of N-(phosphonomethyl) glycine (480 g L^−1^) at a dose of 3.0 L ha^−1^. The solution consisted of water, herbicide, and dye. Before application, a flight plan was prepared for the RPA; according to the plots, range, and application rate, the flight height and displacement speed for autonomous flight were determined. The RPA flights were performed back-to-back.

**Figure 4:**
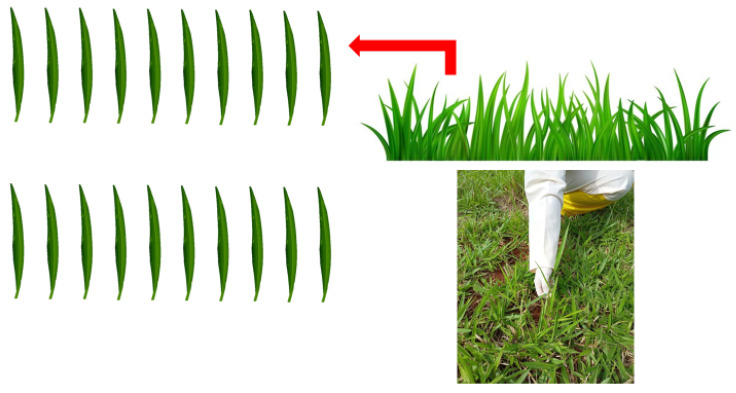
Method of sampling the leaves to assess deposition.

After application, samplings were performed, where twenty (20) leaves of *Urochloa decumbens* were manually collected at random (Figure 4) at eight different points per plot. It should be noted that to collect the leaves, the researchers were equipped with gloves and the collection point was close to the ligule and auricle in order to preserve the tracer in the samples. Furthermore, before collection, a period of drying of the leaves was respected. The leaves were stored, according to each replicate and plot, in previously labelled plastic bags (PVC); the bags were sealed and placed in containers equipped with thermal and light insulation and then transported to the Agricultural Mechanization Laboratory of the Federal University of Uberlândia (Uberlândia, Minas Gerais, Brazil), where the subsequent analyses for residue levels were performed.

The deposited material was removed from the leaf samples in the laboratory. For this, 100 mL of deionized water was added to each plastic bag with the samples. Then, the plastic bags with the solution were shaken for complete homogenization using a pendulum shaker table model TE240/I from the company Tecnal (Piracicaba, Brazil) at 250 rpm for 15 min to extract all the tracer present in the samples.

From the solutions formed, the dye was quantified through the absorbance values obtained by spectrophotometry. The spectrophotometer used in the experiment was a Bioespectro (Curitiba, Brazil) model SP-220 with a tungsten halogen lamp and glass cuvettes with an optical path of 10 mm and a wavelength of 630 nm to detect the colour blue.

To calculate the deposition, the absorbance values were converted into the concentration of tracer in μg L^−1^ using a calibration curve previously determined from solutions with known concentration; then, the mass of tracer deposited was obtained in relation to the amount of extractor solution used to wash the leaves. Subsequently, the tracer mass was divided by the leaf area, in cm^2^, of each sample so that the deposition was determined in μg cm^−2^. Leaf area was determined using an LI 3100C leaf area meter, from Licor^®^ (Lincoln, NE, USA).

#### 3.3.2. Droplet Spectrum

**Figure 5:**
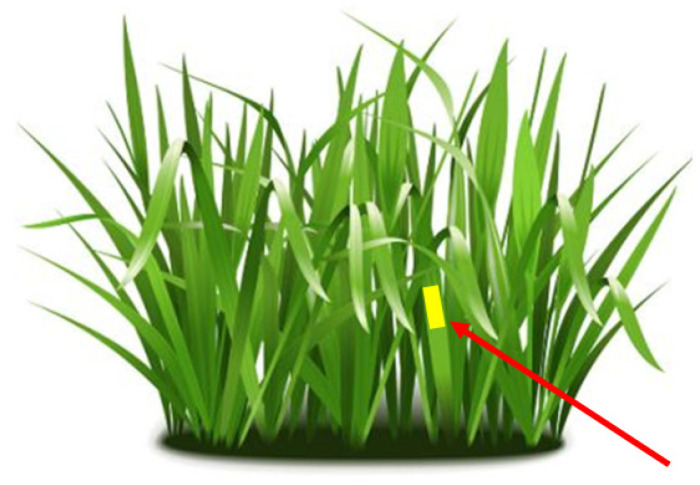
Characterization of water-sensitive paper sampling in the plant.

The droplet spectrum formed by the spraying was characterized by Syngenta (Basel, Switzerland) water-sensitive paper labels with dimensions of 76 × 26 mm. One label was used per repetition within the plots (Figure 5). Metal clips were used to fix the labels to the plants in order to simulate the leaf itself.

Soon after application in each plot, the labels were collected and placed inside duly marked paper envelopes. Then, they were scanned and analysed using the DropScope^®^ system from SprayX (São Carlos, Brazil), equipment that is exclusively for this type of analysis.

The droplet coverage (%), droplet density (droplets cm^−2^), volume median diameter (VMD, μm), relative amplitude (RA), and the percentage of volume in droplets smaller than 100 µm (% < 100 µm) were obtained.

#### 3.3.3. Effectiveness in Controlling *Urochloa decumbens*

The aerial part of the plants was monitored, observing the morphological changes, at 14, 21, and 28 days after application (DAA), where the control efficiency was determined in relation to the day that the application was performed. This analysis was performed visually with a percentage score, based on the methodology by Chen et al. [69], according to Table 6. The control was evaluated at eight points (per replication) within the useful area of the plot.

### 3.4. Statistical Analyses

In this study, the assumptions of the linear model were tested. The Shapiro–Wilk (W) test was used to test the normality of the residuals. Levene’s test (L) was used to test the homogeneity of the variance. The Durbin–Watson test (DW) was used to assess the independence of residuals. If these assumptions were satisfied, an analysis of variance study was performed for a CRD with the four treatments.

Data transformations were used when linear model assumptions were not satisfied. For this, the square root and logarithmic (ln) transformations were used. If after transformation, the assumptions were still not satisfied, nonparametric statistics were used, such as the Kruskal–Wallis test [70], where the analysis is based on a comparison between the medians of the groups. This represents the measure of data position based on the medians, due to nonnormality.

To assess whether the spray solution deposition process within each plot was within the acceptable variability, i.e., to verify the deposition quality, control graphs were prepared using statistical process control. In the construction of the individual control graphs, the individual measurements of each treatment were used, and for the control graphs of the variability between two consecutive measurements, the moving amplitude was used, according to Montgomery [71].

The analyses were performed using the software R version 4.2.2 [72], and control charts were prepared in the software Minitab^®^ version 16.2 [73].

## 4. Conclusions

The application of glyphosate via RPA provided higher tracer deposition on the target and similar effectiveness in controlling *Urochloa decumbens* compared to ground application, proving to be a technically viable option and even resulting in lower droplet coverage and density.

The XR 11001 nozzle was more effective in controlling *Urochloa decumbens* and droplet density. However, it yielded a droplet spectrum with lower VMD and higher volume percentage of droplets smaller than 100 μm, which implies a greater risk of drift.

The AirMix 11001 nozzle was less effective in controlling *Urochloa decumbens* than the XR 11001, but the level of control was equal to or greater than 85% from 21 DAA onwards. Furthermore, the performance of this nozzle regarding deposition, coverage, and relative amplitude was similar to that of the XR 11001. In addition, the percentage of the droplet volume smaller than 100 μm for the AirMix 11001 was less than 1%, also qualifying this nozzle for use in RPA and ground applications, due to its good performance, especially with regard to the risk of drift.

Regarding the spray solution deposition, all applications showed random variability, being indifferent to the process, i.e., the process remained stable, under control, and with the same quality standard.

## Figures and Tables

**Table 1:** Medians of tracer deposition (μg cm^−2^) obtained by the application of a herbicide mixture (glyphosate) via different methods and with the use of two different nozzles for the control of *Urochloa decumbens*.

Application	Deposition (μg cm^−2^)
AERIAL-RPA ^1^	3.466 a
GROUND	2.242 b
Nozzle	
XR 11001	2.919 a
AIRMIX 11001	2.372 a
Assumptions	**W = 0.96**; L= 9.41; **DW = 2.00**

Medians followed by different lowercase letters in the column differ from each other according to the Kruskal–Wallis test at the 0.05 level of significance; W, L, and DW: Shapiro–Wilk statistics for normality of residues, Levene’s test for homogeneity of variances, and Durbin–Watson test for independence of residues, respectively; values in bold indicate normally distributed and independent residues and homogeneous variances at the 0.05 significance level; ^1^ RPA: remotely piloted aircraft.

**Table 2:** Mean coverage (%), droplet density (droplets cm^−2^), and relative amplitude (RA) obtained by the application of herbicide spray (glyphosate) via different methods and with the use of two nozzles for the control of *Urochloa decumbens*.

Application	Coverage (%)	Density ^1^ (Droplets cm^−2^)	Relative Amplitude (RA)
AIR-RPA	5.05 b	55.43 b	0.98 b
GROUND	18.14 a	239.88 a	1.32 a
Nozzle			
XR 11001	10.65 a	213.89 a	1.08 a
AIRMIX 11001	12.54 a	81.41 b	1.21 a
Assumptions	**W = 0.96; L = 2.06; DW = 2.03**	**W = 0.99; L = 2.43; DW = 2.13**	**W = 0.95; L = 0.82; DW = 1.80**

Means followed by different lowercase letters in the columns differ from each other according to Tukey’s test at the 0.05 significance level; W, L, and DW: Shapiro–Wilk statistics for normality of residues, Levene’s test for homogeneity of variances, and Durbin–Watson test for independence of residues, respectively; values in bold indicate normally distributed and independent residues and homogeneous variances at the 0.05 significance level; ^1^ logarithmic transformation; RPA: remotely piloted aircraft.

**Table 3:** Mean volume median diameter (VMD, μm) and percentage of the volume composed of droplets with a diameter smaller than 100 μm (% < 100 μm, %), obtained by the application of a herbicide solution (glyphosate) via different methods and with the use of two nozzles for the control of *Urochloa decumbens*.

	VDM (μm) ^1^	
	XR 11001	AIRMIX 11001
AERIAL APPLICATION-RPA	259.81 aB	393.38 bA
ground APPLICATION	230.97 aB	435.99 aA
Assumptions	**W = 0.97**; **L = 1.09**; **DW = 2.42**	
	**% < 100 μm ^1,2^**	
	XR 11001	AIRMIX 11001
AERIAL APPLICATION-RPA	2.44 bA	0.81 aB
ground APPLICATION	4.08 aA	0.54 aB
Assumptions	**W = 0.98**; **L = 2.11**; **DW = 1.74**	

Means followed by distinct lowercase letters in the columns and uppercase letters in the rows differ from each other according to Tukey’s test at the 0.05 level of significance; W, L, and DW: Shapiro–Wilk statistics for normality of residuals, Levene’s test for homogeneity of variances, and Durbin-Watson test for independence of residuals, respectively; values in bold indicate normally distributed and independent residues and homogeneous variances at the 0.05 significance level; ^1^ significant interaction between factors; ^2^ square-root transformation; RPA: remotely piloted aircraft.

**Table 4:** Percentage of effectiveness in the control of *Urochloa decumbens* from the application of herbicide solution (glyphosate) by different methods and using different nozzles at 14, 21, and 28 days after application (DAA).

	Control (%)		
Application	14 DAA ^1^	21 DAA ^2^	28 DAA ^2^
AIR-RPA	77.19 a	93.00 a	90.00 a
GROUND	75.50 a	95.00 a	88.00 a
Nozzle			
XR 11001	78.06 a	95.00 a	90.00 a
AIRMIX 11001	74.62 b	90.00 b	85.00 b
Assumptions	**W = 0.96**; **L = 0.35**; **DW = 2.07**	W = 0.91; **L = 0.33**; **DW = 2.57**	W = 0.93; **L = 0.20**; **DW = 1.68**

^1^ Means followed by distinct lowercase letters in the column differ from each other according to Tukey’s test at the 0.05 significance level; ^2^ medians followed by distinct lowercase letters in the column differ from each other according to the Kruskal–Wallis test at the 0.05 significance level; W, L, and DW: Shapiro–Wilk statistics for normality of residues, Levene’s test for homogeneity of variances, and Durbin–Watson test for independence of residues, respectively; values in bold indicate normally distributed and independent residues and homogeneous variances at the 0.05 significance level; RPA: remotely piloted aircraft.

**Table 5:** Remotely piloted aircraft specifications.

Parameter	Description
Method of operation	Remote control
Dimensions (mm)	1471 × 1471 × 482 (frame arms unfolded)
Work capacity (ha h^−1^)	2.80–4.05
Spraying system	Atomized spraying
Tank capacity (L)	10
Number of nozzles	4
Application range (m)	4–6 (with application 1.5–3.0 m from the crop)
Accuracy in altitude detection (m)	<0.1
Maximum operating speed (m s^−1^)	8
Positioning mode	GPS ^1^ or manual

^1^ Global positioning system. Source: DJI [68].

**Table 6:** Description of the visual inspection scale as a function of the percentage of weed control effectiveness.

Control (%)	Conceptual Description
100	No surviving individuals of the observed target weed species
95–99	Very good control, with sporadic individuals of the target weed species
85–94	Acceptable control, with a definite decrease in the occurrence of target weeds
70–84	General but insufficient control of the target weeds
60–69	Some control of the target weeds, without commercial value
<60	Poor control of the target weeds, without commercial value

Source: Chen [69].

## Data Availability

Data are contained within the article.
